# Crystal structures of *N*,*N*-dimethyl-(2-(2,2-diphen­yl)-2-prop-2-yn­yloxy)acet­oxy)ethyl­amine and *N*,*N*-dimethyl-(2-(2,2-diphen­yl)-2-prop-2-yn­yl­­oxy)acet­oxy)ethyl­ammonium 2,4,6-tri­nitro­phenolate

**DOI:** 10.1107/S2056989017012968

**Published:** 2017-09-15

**Authors:** Mohammed A. E. Shaibah, Hemmige S. Yathirajan, S. Madan Kumar, Kullaiah Byrappa, Christopher Glidewell

**Affiliations:** a Department of Studies in Chemistry, University of Mysore, Manasagangotri, Mysuru 570 006, India; b Department of Studies in Chemistry, Mangalore University, Mangalagangotri 574 199, India; c Materials Science Center, NCHS Building, University of Mysore, Manasagangotri, Mysuru 570 006, India; d School of Chemistry, University of St Andrews, St Andrews, Fife KY16 9ST, UK

**Keywords:** crystal structure, disorder, mol­ecular conformation, hydrogen bonding

## Abstract

The *N*,*N-*di­methyl­ethylamminium unit in *N*,*N*-dimethyl-[2-(2,2-diphen­yl)-2-prop-2-ynyloxyacet­oxy]ethyl­amine, C_21_H_23_NO_3_ (I), is disordered over two sets of atomic sites having occupancies of 0.880 (3) and 0. 120 (3), but there are no direction-specific inter­actions between the mol­ecules of (I). The cation in *N*,*N*-dimethyl-[2-(2,2-diphen­yl)-2-prop-2-ynyloxyacet­oxy]ethyl­ammonium 2,4,6-tri­nitro­phenolate (picrate), C_21_H_24_NO_3_^+^·C_6_H_2_N_3_O_7_^−^ (II), shows a similar type of disorder, with occupancies of 0.654 (11) and 0.346 (11), although the overall conformation of the cation in (II) is different from that in the neutral (I). The component ions are are linked by an almost planar three-centre N—H⋯(O)_2_ hydrogen bond, and the ion pairs are further linked by a combination of three C—H⋯O hydrogen bonds to form sheets. Comparisons are made with some related structures.

## Abstract 2

The mol­ecule of the neutral title compound and the cation in the title salt both exhibit a similar kind of disorder, even though their overall conformations are different. There are no direction-specific inter­actions between the mol­ecules of the neutral compound, but the ions in the salt are linked into hydrogen-bonded sheets.

## Chemical context   

*N*,*N*-dimethyl-[2-(2,2-diphen­yl)-2-prop-2-ynyloxyacet­oxy]ethyl­amine (pargeverine) is an established anti-spasmodic drug (Mishra *et al.*, 2010 ▸). Although crystal structures have been reported (Bindya *et al.*, 2007 ▸; Harrison, Bindya *et al.*, 2007 ▸; Harrison, Sreevidya *et al.*, 2007 ▸; Swamy *et al.*, 2007 ▸; Yathirajan *et al.*, 2007 ▸; Jasinski *et al.*, 2009 ▸) for a number of related compounds that exhibit a range of pharmacological activities (*e.g.* Matsushima *et al.*, 1997 ▸), the structure of pargeverine itself has not yet been reported. Here we report the structure of the neutral compound (I) and its 2,4,6-tri­nitro­phenolate (picrate) salt (II).

## Structural commentary   

**Figure 1:**
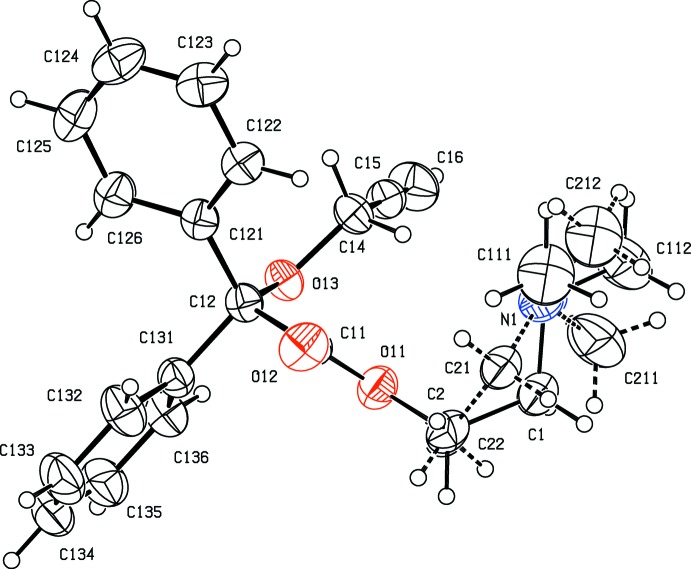
The mol­ecular structure of compound (I) showing the atom-labelling scheme and the disorder. Displacement ellipsoids are drawn at the 30% probability level, and the minor disorder component is drawn with broken lines.

In the neutral compound (I) (Fig. 1 ▸) the methyl­amino­ethyl fragment is disordered over two sets of atomic sites with occupancies of 0.880 (3) for the major disorder component comprising the atomic sites C2,C1,N1,C111 and C112, and 0.120 (3) for the minor component, comprising the atomic sites C22,C21,N21,C211 and C212. The atomic sites in the two disorder components exhibit an approximately mirror-image relationship, as shown by the corresponding pairs of torsional angles, thus: O11—C2—C1—N1 = 59.5 (5)° and O11—C22—C21—N21 = −57 (3)°, C2—C1—N1—C111 = 68.9 (4)° and C22—C21—N21—C211 = −56 (2)°, and C2—C1—N1—C112 = −167.3 (4)° and C22—C21—N21—C212 = −180 (2)°. Exact, though nonetheless non-crystallographic symmetry, would require that the corresponding torsional angles have identical magnitudes, but opposite signs. An unexpected feature of this conformational disorder is the close proximity of the two sites N1 and N21, which are separated by only 0.182 (18) Å.
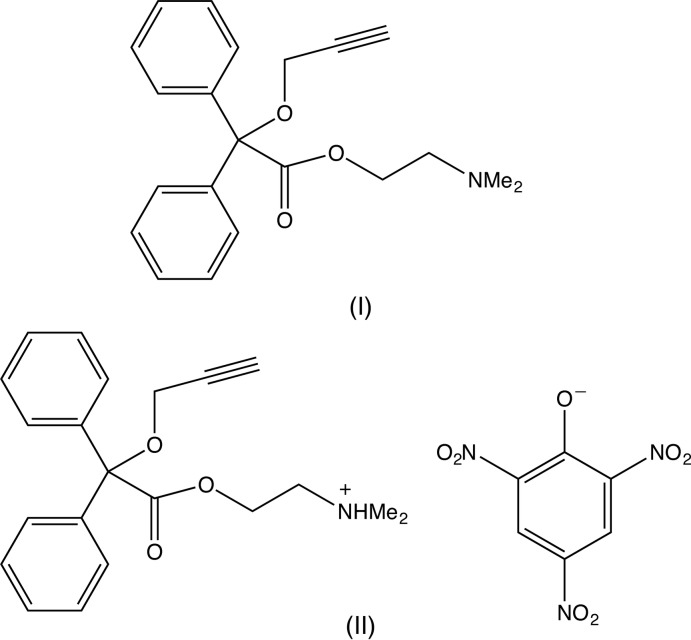


**Figure 2:**
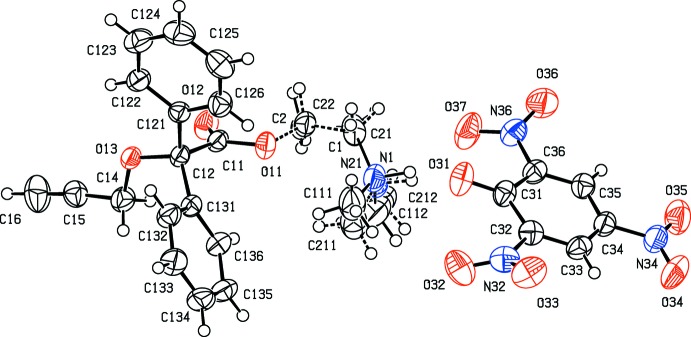
The ionic components of compound (II) showing the atom-labelling scheme and the disorder. Displacement ellipsoids are drawn at the 30% probability level, and the minor disorder component is drawn with broken lines.

In the cation of the picrate salt (II) (Fig. 2 ▸), the same fragment is disordered, again over two sets of atomic sites, but now with occupancies of 0.654 (11) and 0.346 (11). The physical separation of the two sets of atomic sites is, in general, rather less in (II) than in (I), but the overall conformation of the cation in (II) is different from that of the neutral compound (I). This is well illustrated by the values of the torsion angles O12—C11—C12—O13, 157.8 (2)° in (I) and 13.1 (2)° in (II), and C11—O11—C2—C1 − 123.1 (4)° in (I) and 172.8 (4)° in (II), resulting in very different locations for the disordered fragment relative to the fragment Ph_2_COCH_2_CCH (*cf*. Figs. 1 ▸ and 2 ▸).

The C—O distance in the picrate anion in (II), 1.2486 (17) Å, is short for its type [mean value (Allen *et al.*, 1987 ▸) 1.362 Å, lower quartile value 1.353 Å]; the C—N distances in this anion, in the range 1.445 (2)–1.459 (2) Å, all fall below the mean value of 1.468 Å for bonds of this type. In addition, the C31—C32 and C31—C36 distances are 1.445 (2) and 1.439 (2) Å, respectively, whereas the other four C—C distances in this ring lie in the range 1.367 (2)–1.385 (2) Å with a mean value of 1.375 Å. These observations point to significant contributions to the electronic structure of this anion of polarized forms in which the negative charge is delocalized from the phenolic O atom into the ring and thence onto the nitro groups as recently noted (Sagar *et al.*, 2017 ▸).

## Supra­molecular features   

Despite the abundance of potential hydrogen-bond donors and acceptors in (I), with the C—H bonds of the aryl rings and the alkynyl unit as potential donors, and the amino N atom, the carbonyl O atom, two aryl rings and the triple bond of the alkynyl function as potential acceptors, there are in fact, no hydrogen bonds of any kind in the crystal structure of (I): nor are there any aromatic π–π stacking inter­actions, so that the structure consists of essentially isolated mol­ecules making only van der Waals-type contacts with one another.

**Figure 3:**
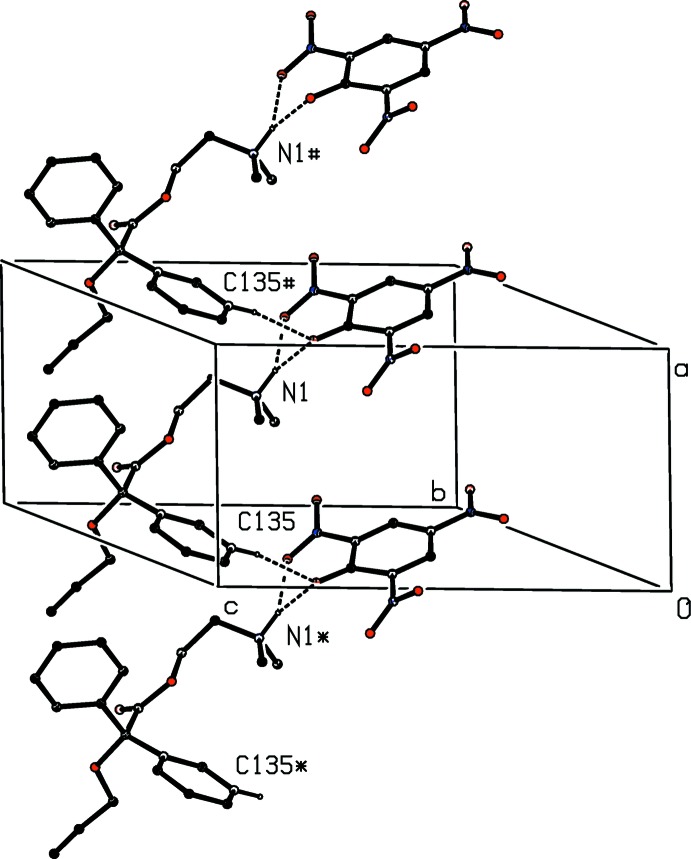
Part of the crystal structure of compound (II) showing the formation of a hydrogen-bonded chain running parallel to [100]. For the sake of clarity, only the major disorder component of the cation is shown and the H atoms not involved in the motif shown have been omitted. The atoms marked with an asterisk (*) and a hash (#) are at the symmetry positions (−1 + *x*, *y*, *z*) and (1 + *x*, *y*, *z*), respectively.

**Figure 4:**
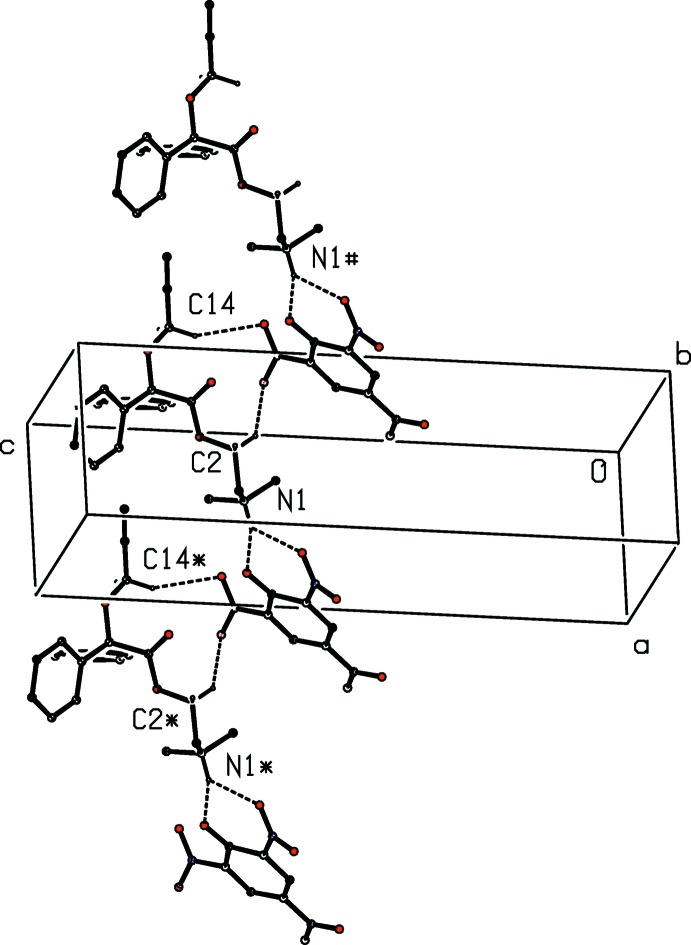
Part of the crystal structure of compound (II) showing the formation of a hydrogen-bonded chain of rings running parallel to [1
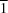
0]. For the sake of clarity, only the major disorder component of the cation is shown and the H atoms bonded to the C atoms which are not involved in the motif shown have been omitted. The atoms marked with an asterisk (*) and a hash (#)are at the symmetry positions (1 + *x*, −1 + *y*, *z*) and (−1 + *x*, 1 + *y*, *z*), respectively.

Both disorder components of the cation in (II) are linked to the anion within the selected asymmetric unit *via* a near planar, but markedly asymmetric three-centre N-H⋯(O)_2_ charge-assisted (Gilli *et al.*, 1994 ▸) hydrogen bond (Table 1 ▸), which forms an 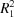
(6) motif. The resulting ion pairs are further linked by three C—H⋯O hydrogen bonds into complex sheets: however, the straightforward identification of two simple one-dimensional sub-structures (Ferguson *et al.*, 1998*a* ▸,*b* ▸; Gregson *et al.*, 2000 ▸) leads to a simple analysis of the sheet formation. In the simpler of the two sub-structures, the C—H⋯O hydrogen bond involving an aryl C—H unit links ion pairs related by translation along [100] into a 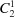
(12) chain (Fig. 3 ▸). In the second sub-structure, the cooperative effect of two C—H⋯O hydrogen bonds, both involving CH_2_ groups, generates a chain parallel to [1
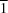
0] containing alternating 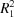
(6) and 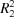
(11) rings (Fig. 4 ▸). The combination of these two chain motifs generates a sheet lying parallel to (001) in the domain 0.5 < *z* < 1.0: a second such sheet, related to the first by inversion, lies in the domain 0 < *z* < 0.5, but there are no direction-specific inter­actions between adjacent sheets.

## Database survey   

In the (2*R*,3*R*)-(hydrogentartrate) salt (III) (Glidewell *et al.*, 2017 ▸), the cation is fully ordered, unlike that in the picrate (II) and the conformation of the cation closely resembles that of the neutral mol­ecule (I).
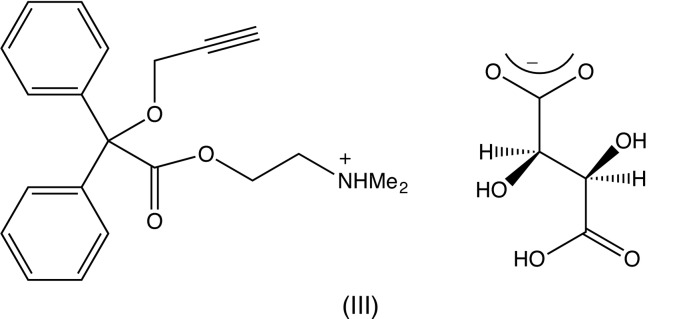


**Figure 5:**
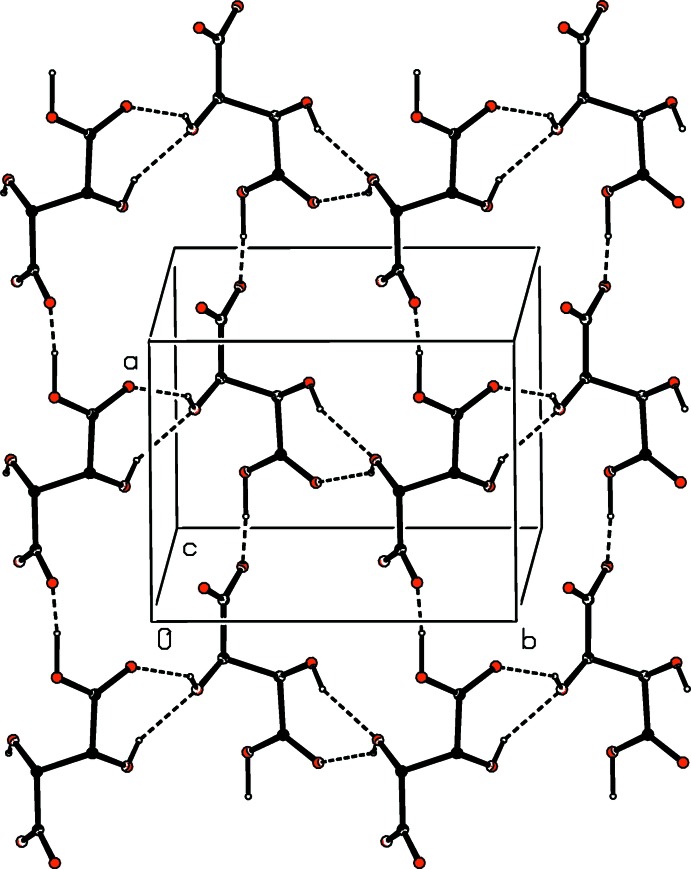
Part of the crystal structure of compound (III) showing the formation of a hydrogen-bonded sheet of anions parallel to (001). The original atomic coordinates (Glidewell *et al.*, 2017 ▸) have been used and, for the sake of clarity, the H atoms bonded to C atoms have been omitted.

**Figure 6:**
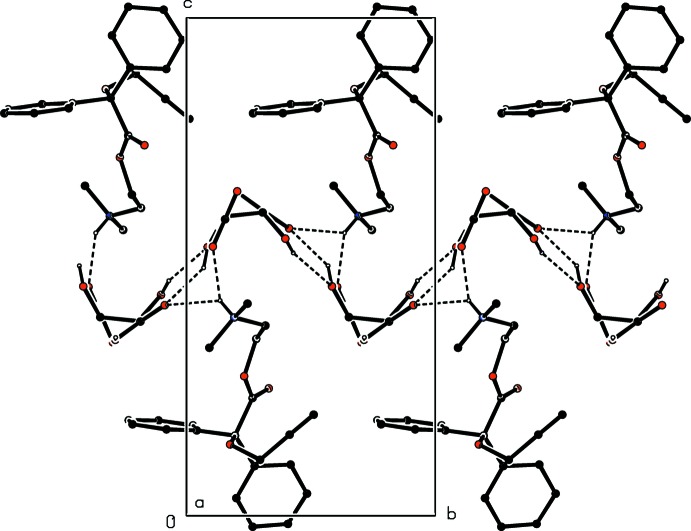
A projection down [100] of part of the crystal structure of compound (III) showing the disposition of the cations bonded to both faces of the anion sheet. For the sake of clarity, the H atoms bonded to C atoms have been omitted.

The anions are linked by three O—H⋯O hydrogen bonds to form sheets lying parallel to (001) and containing equal numbers of 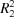
(7) and 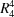
(21) rings (Fig. 5 ▸). Within this sheet, the anions related by translation along [100] are linked by a very short and nearly linear O—H⋯O hydrogen bond, although the H atom is nonetheless off-centre; O⋯O^i^ 2.461 (7) Å; O—H⋯O^i^ 167 (9)°, O—H 1.12 (1) Å, H⋯O^i^ 1.35 (10) Å [symmetry code: (i) 1 + *x*, *y*, *z*]. The cations are linked to this sheet by a three-centre N—O⋯(O)_2_ hydrogen bond and they are disposed to either side of the sheet (Fig. 6 ▸).

4-(2,2-Diphenyl-2-propoxyacet­oxy)-1-methyl­piperidin-1-ium picrate (propiverinium picrate) (IV) is closely related to compound (II), differing in containing a saturated alk­oxy substituent and having an *N*-methyl piperidinium unit in place of the *N*,*N*-di­methyl­ethyl­ammonium unit in (II). The component anions in (IV) are linked (Jasinski *et al.*, 2009 ▸) by the same type of hydrogen-bonded (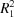
6) ring as seen in (II) but there are no structurally significant inter­actions between adjacent ion pairs in (IV).

## Synthesis and crystallization   

A sample of compound (I) was a gift from RL Fine Chem, Pvt. Ltd., Bengaluru, India, and it was recrystallized from methanol solution by slow evaporation at room temperature, m.p. 347–351 K. For the preparation of compound (II), equimolar qu­anti­ties (0.30 mmol) of (I) and picric acid were dissolved in hot methanol and the solution was held at 333 K for 0.5 h, with magnetic stirring throughout. The solution was then allowed to cool slowly to room temperature, giving crystals of (II) suitable for single-crystal X-ray diffraction. m.p. 386–389 K.

## Refinement   

Crystal data, data collection and structure refinement details are summarized in Table 2 ▸. It was apparent from an early stage in the refinements that in both (I) and (II) the di­methyl­amino­ethyl portion was disordered over two sets of atomic sites having different occupancies in each case, and corres­ponding to different conformations. For the minor conformation of each compound, the bonded distances and the 1,3-non-bonded distances were restrained to be the same as the corresponding distances in the major conformer, subject to s.u.s of 0.005 and 0.01 Å, respectively: in addition, the anisotropic displacement parameters for corresponding pairs of atomic sites occupying essentially the same physical space were constrained to be equal. All H atoms, other than those in the minor disorder components, were located in difference maps, and then treated as riding atoms in geometrically idealized position, with distances C—H 0.93 Å (aromatic and alkyne), 0.96 Å (CH_3_) or 0.97 Å (CH_2_) and N—H 0.98 Å, with *U*_iso_(H) = *kU*_eq_(carrier), where *k* = 1.5 for the methyl groups, which were permitted to rotate but not to tilt, and 1.2 for all other H atoms. The H atoms in the minor disorder components were included in calculated positions using the same procedure. When the refinement of the atomic coordinates for the H atoms bonded to N atoms in (II) was attempted, the resulting N—H distances were 1.04 (4) and 0.82 (8) Å: accordingly, the riding model was preferred. Two low-angle reflections which had been attenuated by the beam stop, (020) for (I) and (002) for (II), were omitted from the final refinements. Subject to these conditions, the occupancies of the disorder components were 0.880 (3) and 0.120 (3) in (I) and 0.654 (11) and 0.346 (11) in (II). In the final analyses of variance for (I) there was a large value, 22.969, of *K* = [mean(*F*_o_^2^)/mean(*F*_c_^2^)] for the group of 518 very weak reflections having *F*_c_/*F*_c_(max) in the range 0.000 < *F*_c_/*F*_c_(max) < 0.004, and for (II) a value of *K* = 9.509 for the group of 789 very weak reflections having *F*_c_/*F*_c_(max) in the range 0.000 < *F*_c_/*F*_c_(max) < 0.006.

## Supplementary Material

Crystal structure: contains datablock(s) global, I, II. DOI: 10.1107/S2056989017012968/bq2403sup1.cif

Structure factors: contains datablock(s) I. DOI: 10.1107/S2056989017012968/bq2403Isup2.hkl

Structure factors: contains datablock(s) II. DOI: 10.1107/S2056989017012968/bq2403IIsup3.hkl

CCDC references: 1562193, 1562192

Additional supporting information:  crystallographic information; 3D view; checkCIF report

## Figures and Tables

**Table 1:** Hydrogen-bond geometry (Å, °) for (II)

*D*—H⋯*A*	*D*—H	H⋯*A*	*D*⋯*A*	*D*—H⋯*A*
N1—H1⋯O31	0.98	1.72	2.633 (7)	153
N1—H1⋯O37	0.98	2.33	3.010 (7)	126
N21—H21⋯O31	0.98	1.77	2.697 (14)	157
N21—H21⋯O37	0.98	2.34	2.944 (14)	120
C2—H2*B*⋯O33^i^	0.97	2.56	3.338 (9)	137
C14—H14*B*⋯O32^i^	0.97	2.52	3.407 (2)	153
C135—H135⋯O31^ii^	0.93	2.58	3.485 (2)	165

Symmetry codes: (i) 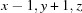; (ii) 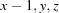.

**Table 2:** Experimental details

	(I)	(II)
Crystal data		
Chemical formula	C_21_H_23_NO_3_	C_21_H_24_NO_3_·C_6_H_2_N_3_O_7_
*M*_r_	337.40	566.52
Crystal system, space group	Monoclinic, *P*2_1_/*n*	Triclinic, *P* 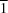
Temperature (K)	296	296
*a*, *b*, *c* (Å)	9.2545 (9), 21.7246 (19), 9.4531 (9)	7.5208 (3), 8.3919 (3), 22.2282 (7)
α, β, γ (°)	90, 94.763 (9), 90	85.099 (3), 84.294 (3), 75.117 (3)
*V* (Å^3^)	1894.0 (3)	1346.51 (9)
*Z*	4	2
Radiation type	Mo *K*α	Mo *K*α
μ (mm^−1^)	0.08	0.11
Crystal size (mm)	0.30 × 0.24 × 0.23	0.23 × 0.21 × 0.21

Data collection		
Diffractometer	Rigaku Saturn724	Rigaku Saturn724
Absorption correction	Multi-scan (*SADABS*; Sheldrick,2003 ▸)	Multi-scan (*SADABS*; Sheldrick,2003 ▸)
*T*_min_, *T*_max_	0.956, 0.982	0.949, 0.978
No. of measured, independent and observed [*I* > 2σ(*I*)] reflections	23094, 4348, 2221	18671, 7547, 4973
*R*_int_	0.066	0.033
(sin θ/λ)_max_ (Å^−1^)	0.651	0.728

Refinement		
*R*[*F* ^2^ > 2σ(*F* ^2^)], *wR*(*F* ^2^), *S*	0.064, 0.195, 1.04	0.053, 0.136, 1.05
No. of reflections	4348	7547
No. of parameters	247	390
No. of restraints	10	10
H-atom treatment	H-atom parameters constrained	H-atom parameters constrained
Δρ_max_, Δρ_min_ (e Å^−3^)	0.16, −0.15	0.27, −0.22

Computer programs: *CrystalClear* (Rigaku, 2011 ▸), *SHELXS86* (Sheldrick, 2008 ▸), *SHELXL2014* (Sheldrick, 2015 ▸) and *PLATON* (Spek, 2009 ▸).

## supplementary crystallographic information

### N,N-Dimethyl-[2-(2,2-diphenyl)-2-prop-2-ynyloxyacetoxy]ethylamine (I) Crystal data

**Table:**

C_21_H_23_NO_3_	*F*(000) = 720
*M**_r_* = 337.40	*D*_x_ = 1.183 Mg m^−^^3^
Monoclinic, *P*2_1_/*n*	Mo *K*α radiation, λ = 0.71073 Å
*a* = 9.2545 (9) Å	Cell parameters from 5622 reflections
*b* = 21.7246 (19) Å	θ = 1.9–31.2°
*c* = 9.4531 (9) Å	µ = 0.08 mm^−^^1^
β = 94.763 (9)°	*T* = 296 K
*V* = 1894.0 (3) Å^3^	Block, colourless
*Z* = 4	0.30 × 0.24 × 0.23 mm

### N,N-Dimethyl-[2-(2,2-diphenyl)-2-prop-2-ynyloxyacetoxy]ethylamine (I) Data collection

**Table:**

Rigaku Saturn724 diffractometer	2221 reflections with *I* > 2σ(*I*)
Radiation source: fine focus sealed tube	*R*_int_ = 0.066
φ and ω scans	θ_max_ = 27.6°, θ_min_ = 2.4°
Absorption correction: multi-scan (SADABS; Sheldrick,2003)	*h* = −9→11
*T*_min_ = 0.956, *T*_max_ = 0.982	*k* = −28→28
23094 measured reflections	*l* = −12→12
4348 independent reflections	

### N,N-Dimethyl-[2-(2,2-diphenyl)-2-prop-2-ynyloxyacetoxy]ethylamine (I) Refinement

**Table:**

Refinement on *F*^2^	Hydrogen site location: inferred from neighbouring sites
Least-squares matrix: full	H-atom parameters constrained
*R*[*F*^2^ > 2σ(*F*^2^)] = 0.064	*w* = 1/[σ^2^(*F*_o_^2^) + (0.0758*P*)^2^ + 0.2089*P*] where *P* = (*F*_o_^2^ + 2*F*_c_^2^)/3
*wR*(*F*^2^) = 0.195	(Δ/σ)_max_ < 0.001
*S* = 1.04	Δρ_max_ = 0.16 e Å^−^^3^
4348 reflections	Δρ_min_ = −0.15 e Å^−^^3^
247 parameters	Extinction correction: SHELXL, Fc^*^=kFc[1+0.001xFc^2^λ^3^/sin(2θ)]^-1/4^
10 restraints	Extinction coefficient: 0.011 (2)

### N,N-Dimethyl-[2-(2,2-diphenyl)-2-prop-2-ynyloxyacetoxy]ethylamine (I) Special details

**Table:**

Geometry. All esds (except the esd in the dihedral angle between two l.s. planes) are estimated using the full covariance matrix. The cell esds are taken into account individually in the estimation of esds in distances, angles and torsion angles; correlations between esds in cell parameters are only used when they are defined by crystal symmetry. An approximate (isotropic) treatment of cell esds is used for estimating esds involving l.s. planes.

### N,N-Dimethyl-[2-(2,2-diphenyl)-2-prop-2-ynyloxyacetoxy]ethylamine (I) Fractional atomic coordinates and isotropic or equivalent isotropic displacement parameters (Å^2^)

**Table:**

	*x*	*y*	*z*	*U*_iso_*/*U*_eq_	Occ. (<1)
N1	0.1191 (3)	0.41234 (15)	0.8098 (3)	0.0714 (6)	0.880 (3)
C111	0.2407 (5)	0.37948 (19)	0.7606 (5)	0.1196 (16)	0.880 (3)
H11A	0.3275	0.4032	0.7805	0.179*	0.880 (3)
H11B	0.2247	0.3728	0.6602	0.179*	0.880 (3)
H11C	0.2509	0.3405	0.8084	0.179*	0.880 (3)
C112	−0.0119 (5)	0.3773 (2)	0.7788 (5)	0.1315 (16)	0.880 (3)
H11D	−0.0068	0.3400	0.8336	0.197*	0.880 (3)
H11E	−0.0224	0.3673	0.6796	0.197*	0.880 (3)
H11F	−0.0937	0.4011	0.8026	0.197*	0.880 (3)
C1	0.1396 (3)	0.42760 (13)	0.9570 (3)	0.0735 (9)	0.880 (3)
H1A	0.1655	0.3906	1.0107	0.088*	0.880 (3)
H1B	0.0488	0.4427	0.9880	0.088*	0.880 (3)
C2	0.2551 (7)	0.47531 (13)	0.9896 (4)	0.0849 (9)	0.880 (3)
H2A	0.2646	0.4840	1.0906	0.102*	0.880 (3)
H2B	0.3476	0.4602	0.9627	0.102*	0.880 (3)
O11	0.21492 (17)	0.53133 (7)	0.91034 (17)	0.0686 (5)	0.880 (3)
N21	0.1334 (19)	0.4120 (9)	0.8101 (19)	0.0714 (6)	0.120 (3)
C212	0.143 (3)	0.3597 (14)	0.718 (3)	0.1196 (16)	0.120 (3)
H21A	0.2189	0.3663	0.6562	0.179*	0.120 (3)
H21B	0.0523	0.3545	0.6614	0.179*	0.120 (3)
H21C	0.1634	0.3233	0.7734	0.179*	0.120 (3)
C211	0.012 (2)	0.4041 (15)	0.895 (3)	0.1315 (16)	0.120 (3)
H21D	−0.0293	0.3640	0.8779	0.197*	0.120 (3)
H21E	−0.0593	0.4350	0.8703	0.197*	0.120 (3)
H21F	0.0452	0.4079	0.9938	0.197*	0.120 (3)
C21	0.2645 (18)	0.4226 (5)	0.897 (2)	0.0735 (9)	0.120 (3)
H21G	0.3427	0.4295	0.8369	0.088*	0.120 (3)
H21H	0.2879	0.3861	0.9536	0.088*	0.120 (3)
C22	0.255 (5)	0.4768 (5)	0.9950 (18)	0.0849 (9)	0.120 (3)
H22A	0.1833	0.4690	1.0619	0.102*	0.120 (3)
H22B	0.3482	0.4833	1.0483	0.102*	0.120 (3)
O21	0.21492 (17)	0.53133 (7)	0.91034 (17)	0.0686 (5)	0.120 (3)
C11	0.3078 (3)	0.55286 (10)	0.8243 (2)	0.0560 (6)	
O12	0.42714 (18)	0.53231 (8)	0.81288 (19)	0.0774 (6)	
C12	0.2500 (2)	0.61174 (9)	0.7481 (2)	0.0519 (5)	
O13	0.09551 (14)	0.61353 (6)	0.73420 (15)	0.0549 (4)	
C14	0.0256 (2)	0.56650 (10)	0.6483 (2)	0.0603 (6)	
H14A	0.0515	0.5263	0.6874	0.072*	
H14B	0.0563	0.5686	0.5528	0.072*	
C15	−0.1296 (3)	0.57546 (11)	0.6453 (2)	0.0603 (6)	
C16	−0.2545 (3)	0.58165 (14)	0.6416 (3)	0.0869 (9)	
H16	−0.3545	0.5866	0.6386	0.104*	
C121	0.3093 (2)	0.61909 (10)	0.6028 (2)	0.0538 (6)	
C122	0.3424 (2)	0.56994 (11)	0.5191 (3)	0.0654 (7)	
H122	0.3367	0.5301	0.5543	0.079*	
C123	0.3841 (3)	0.57906 (14)	0.3833 (3)	0.0804 (8)	
H123	0.4062	0.5454	0.3283	0.096*	
C124	0.3928 (3)	0.63714 (17)	0.3298 (3)	0.0898 (9)	
H124	0.4213	0.6431	0.2388	0.108*	
C125	0.3594 (3)	0.68648 (15)	0.4108 (3)	0.0889 (9)	
H125	0.3638	0.7261	0.3740	0.107*	
C126	0.3192 (3)	0.67781 (12)	0.5471 (3)	0.0725 (7)	
H126	0.2986	0.7117	0.6019	0.087*	
C131	0.2994 (2)	0.66400 (10)	0.8497 (2)	0.0571 (6)	
C132	0.4459 (3)	0.67502 (12)	0.8790 (3)	0.0843 (8)	
H132	0.5132	0.6509	0.8365	0.101*	
C133	0.4930 (4)	0.72146 (15)	0.9706 (3)	0.0987 (10)	
H133	0.5918	0.7288	0.9889	0.118*	
C134	0.3965 (4)	0.75646 (13)	1.0342 (3)	0.0952 (10)	
H134	0.4289	0.7876	1.0966	0.114*	
C135	0.2507 (4)	0.74606 (13)	1.0068 (3)	0.0933 (9)	
H135	0.1843	0.7704	1.0498	0.112*	
C136	0.2019 (3)	0.69943 (11)	0.9152 (3)	0.0730 (7)	
H136	0.1030	0.6921	0.8980	0.088*	

### N,N-Dimethyl-[2-(2,2-diphenyl)-2-prop-2-ynyloxyacetoxy]ethylamine (I) Atomic displacement parameters (Å^2^)

**Table:**

	*U*^11^	*U*^22^	*U*^33^	*U*^12^	*U*^13^	*U*^23^
N1	0.0713 (16)	0.0745 (13)	0.0683 (14)	0.0059 (11)	0.0060 (11)	0.0046 (10)
C111	0.122 (4)	0.109 (3)	0.135 (4)	0.016 (3)	0.047 (3)	−0.014 (3)
C112	0.108 (3)	0.154 (4)	0.126 (4)	−0.034 (3)	−0.028 (3)	−0.019 (3)
C1	0.079 (2)	0.0749 (18)	0.0674 (19)	−0.0072 (15)	0.0104 (15)	0.0114 (14)
C2	0.097 (2)	0.0824 (18)	0.0710 (18)	−0.0109 (15)	−0.0182 (15)	0.0298 (14)
O11	0.0646 (11)	0.0706 (10)	0.0701 (11)	−0.0038 (8)	0.0022 (8)	0.0146 (8)
N21	0.0713 (16)	0.0745 (13)	0.0683 (14)	0.0059 (11)	0.0060 (11)	0.0046 (10)
C212	0.122 (4)	0.109 (3)	0.135 (4)	0.016 (3)	0.047 (3)	−0.014 (3)
C211	0.108 (3)	0.154 (4)	0.126 (4)	−0.034 (3)	−0.028 (3)	−0.019 (3)
C21	0.079 (2)	0.0749 (18)	0.0674 (19)	−0.0072 (15)	0.0104 (15)	0.0114 (14)
C22	0.097 (2)	0.0824 (18)	0.0710 (18)	−0.0109 (15)	−0.0182 (15)	0.0298 (14)
O21	0.0646 (11)	0.0706 (10)	0.0701 (11)	−0.0038 (8)	0.0022 (8)	0.0146 (8)
C11	0.0462 (14)	0.0603 (13)	0.0602 (14)	−0.0003 (11)	−0.0033 (11)	−0.0009 (11)
O12	0.0573 (12)	0.0861 (12)	0.0880 (13)	0.0196 (9)	0.0010 (9)	0.0147 (9)
C12	0.0402 (13)	0.0560 (12)	0.0589 (13)	0.0006 (9)	0.0005 (10)	0.0000 (10)
O13	0.0403 (9)	0.0568 (9)	0.0667 (10)	0.0001 (6)	−0.0008 (7)	−0.0052 (7)
C14	0.0474 (14)	0.0655 (14)	0.0664 (15)	−0.0069 (10)	−0.0039 (11)	−0.0062 (11)
C15	0.0486 (15)	0.0732 (15)	0.0586 (14)	−0.0082 (11)	0.0019 (11)	0.0039 (11)
C16	0.0495 (17)	0.114 (2)	0.096 (2)	−0.0066 (15)	0.0000 (14)	−0.0089 (17)
C121	0.0366 (12)	0.0597 (13)	0.0647 (14)	0.0012 (9)	0.0015 (10)	0.0031 (11)
C122	0.0579 (15)	0.0708 (15)	0.0680 (16)	0.0038 (11)	0.0078 (12)	0.0023 (12)
C123	0.0661 (18)	0.104 (2)	0.0729 (18)	0.0116 (15)	0.0140 (13)	−0.0018 (16)
C124	0.072 (2)	0.125 (3)	0.0751 (19)	0.0081 (17)	0.0218 (15)	0.0191 (19)
C125	0.082 (2)	0.090 (2)	0.095 (2)	−0.0066 (16)	0.0172 (17)	0.0297 (18)
C126	0.0689 (17)	0.0704 (16)	0.0783 (18)	0.0006 (12)	0.0058 (13)	0.0116 (13)
C131	0.0549 (15)	0.0526 (12)	0.0626 (14)	−0.0033 (10)	−0.0025 (11)	0.0016 (10)
C132	0.0567 (17)	0.0823 (18)	0.111 (2)	−0.0084 (13)	−0.0133 (15)	−0.0131 (16)
C133	0.087 (2)	0.088 (2)	0.115 (3)	−0.0243 (18)	−0.0298 (19)	−0.0037 (19)
C134	0.133 (3)	0.0632 (17)	0.083 (2)	−0.0200 (19)	−0.029 (2)	−0.0020 (14)
C135	0.115 (3)	0.0709 (18)	0.093 (2)	−0.0019 (17)	0.0038 (19)	−0.0187 (15)
C136	0.0724 (17)	0.0671 (15)	0.0790 (17)	−0.0037 (13)	0.0039 (14)	−0.0103 (13)

### N,N-Dimethyl-[2-(2,2-diphenyl)-2-prop-2-ynyloxyacetoxy]ethylamine (I) Geometric parameters (Å, º)

**Table:**

N1—C1	1.428 (4)	C12—O13	1.426 (2)
N1—C112	1.442 (5)	C12—C121	1.529 (3)
N1—C111	1.442 (4)	C12—C131	1.532 (3)
C111—H11A	0.9600	O13—C14	1.426 (2)
C111—H11B	0.9600	C14—C15	1.447 (3)
C111—H11C	0.9600	C14—H14A	0.9700
C112—H11D	0.9600	C14—H14B	0.9700
C112—H11E	0.9600	C15—C16	1.161 (3)
C112—H11F	0.9600	C16—H16	0.9300
C1—C2	1.502 (6)	C121—C122	1.378 (3)
C1—H1A	0.9700	C121—C126	1.386 (3)
C1—H1B	0.9700	C122—C123	1.385 (3)
C2—O11	1.461 (3)	C122—H122	0.9300
C2—H2A	0.9700	C123—C124	1.364 (4)
C2—H2B	0.9700	C123—H123	0.9300
O11—C11	1.317 (3)	C124—C125	1.367 (4)
N21—C21	1.428 (6)	C124—H124	0.9300
N21—C211	1.441 (7)	C125—C126	1.384 (3)
N21—C212	1.442 (6)	C125—H125	0.9300
C212—H21A	0.9600	C126—H126	0.9300
C212—H21B	0.9600	C131—C136	1.372 (3)
C212—H21C	0.9600	C131—C132	1.382 (3)
C211—H21D	0.9600	C132—C133	1.377 (4)
C211—H21E	0.9600	C132—H132	0.9300
C211—H21F	0.9600	C133—C134	1.352 (4)
C21—C22	1.503 (8)	C133—H133	0.9300
C21—H21G	0.9700	C134—C135	1.371 (4)
C21—H21H	0.9700	C134—H134	0.9300
C22—H22A	0.9700	C135—C136	1.384 (3)
C22—H22B	0.9700	C135—H135	0.9300
C11—O12	1.204 (2)	C136—H136	0.9300
C11—C12	1.542 (3)		
			
C1—N1—C112	111.3 (3)	O12—C11—C12	123.4 (2)
C1—N1—C111	112.7 (3)	O11—C11—C12	111.37 (19)
C112—N1—C111	109.8 (4)	O13—C12—C121	110.28 (16)
N1—C111—H11A	109.5	O13—C12—C131	106.39 (16)
N1—C111—H11B	109.5	C121—C12—C131	112.03 (17)
H11A—C111—H11B	109.5	O13—C12—C11	111.83 (17)
N1—C111—H11C	109.5	C121—C12—C11	111.72 (17)
H11A—C111—H11C	109.5	C131—C12—C11	104.35 (17)
H11B—C111—H11C	109.5	C12—O13—C14	115.91 (15)
N1—C112—H11D	109.5	O13—C14—C15	108.50 (18)
N1—C112—H11E	109.5	O13—C14—H14A	110.0
H11D—C112—H11E	109.5	C15—C14—H14A	110.0
N1—C112—H11F	109.5	O13—C14—H14B	110.0
H11D—C112—H11F	109.5	C15—C14—H14B	110.0
H11E—C112—H11F	109.5	H14A—C14—H14B	108.4
N1—C1—C2	113.3 (3)	C16—C15—C14	178.7 (3)
N1—C1—H1A	108.9	C15—C16—H16	180.0
C2—C1—H1A	108.9	C122—C121—C126	118.0 (2)
N1—C1—H1B	108.9	C122—C121—C12	123.23 (19)
C2—C1—H1B	108.9	C126—C121—C12	118.5 (2)
H1A—C1—H1B	107.7	C121—C122—C123	120.9 (2)
O11—C2—C1	108.8 (3)	C121—C122—H122	119.6
O11—C2—H2A	109.9	C123—C122—H122	119.6
C1—C2—H2A	109.9	C124—C123—C122	120.4 (3)
O11—C2—H2B	109.9	C124—C123—H123	119.8
C1—C2—H2B	109.9	C122—C123—H123	119.8
H2A—C2—H2B	108.3	C123—C124—C125	119.6 (3)
C11—O11—C2	117.4 (3)	C123—C124—H124	120.2
C21—N21—C211	111.1 (8)	C125—C124—H124	120.2
C21—N21—C212	112.7 (8)	C124—C125—C126	120.4 (3)
C211—N21—C212	109.4 (8)	C124—C125—H125	119.8
N21—C212—H21A	109.5	C126—C125—H125	119.8
N21—C212—H21B	109.5	C125—C126—C121	120.7 (3)
H21A—C212—H21B	109.5	C125—C126—H126	119.6
N21—C212—H21C	109.5	C121—C126—H126	119.6
H21A—C212—H21C	109.5	C136—C131—C132	118.9 (2)
H21B—C212—H21C	109.5	C136—C131—C12	121.7 (2)
N21—C211—H21D	109.5	C132—C131—C12	119.4 (2)
N21—C211—H21E	109.5	C133—C132—C131	120.5 (3)
H21D—C211—H21E	109.5	C133—C132—H132	119.8
N21—C211—H21F	109.5	C131—C132—H132	119.8
H21D—C211—H21F	109.5	C134—C133—C132	120.4 (3)
H21E—C211—H21F	109.5	C134—C133—H133	119.8
N21—C21—C22	113.1 (9)	C132—C133—H133	119.8
N21—C21—H21G	109.0	C133—C134—C135	120.0 (3)
C22—C21—H21G	109.0	C133—C134—H134	120.0
N21—C21—H21H	109.0	C135—C134—H134	120.0
C22—C21—H21H	109.0	C134—C135—C136	120.2 (3)
H21G—C21—H21H	107.8	C134—C135—H135	119.9
C21—C22—H22A	109.9	C136—C135—H135	119.9
C21—C22—H22B	109.9	C131—C136—C135	120.0 (3)
H22A—C22—H22B	108.3	C131—C136—H136	120.0
O12—C11—O11	125.0 (2)	C135—C136—H136	120.0
			
C112—N1—C1—C2	−167.3 (4)	C11—C12—C121—C126	−153.9 (2)
C111—N1—C1—C2	68.9 (4)	C126—C121—C122—C123	−0.1 (3)
N1—C1—C2—O11	59.5 (5)	C12—C121—C122—C123	174.4 (2)
C1—C2—O11—C11	−123.1 (4)	C121—C122—C123—C124	−0.1 (4)
C211—N21—C21—C22	−56 (2)	C122—C123—C124—C125	−0.3 (4)
C212—N21—C21—C22	−180 (2)	C123—C124—C125—C126	1.0 (5)
C2—O11—C11—O12	−5.2 (3)	C124—C125—C126—C121	−1.3 (4)
C2—O11—C11—C12	179.8 (2)	C122—C121—C126—C125	0.8 (4)
O12—C11—C12—O13	157.8 (2)	C12—C121—C126—C125	−174.0 (2)
O11—C11—C12—O13	−27.0 (2)	O13—C12—C131—C136	4.0 (3)
O12—C11—C12—C121	33.6 (3)	C121—C12—C131—C136	124.6 (2)
O11—C11—C12—C121	−151.20 (18)	C11—C12—C131—C136	−114.3 (2)
O12—C11—C12—C131	−87.6 (3)	O13—C12—C131—C132	−177.5 (2)
O11—C11—C12—C131	87.6 (2)	C121—C12—C131—C132	−56.9 (3)
C121—C12—O13—C14	61.1 (2)	C11—C12—C131—C132	64.2 (3)
C131—C12—O13—C14	−177.23 (17)	C136—C131—C132—C133	−1.0 (4)
C11—C12—O13—C14	−63.9 (2)	C12—C131—C132—C133	−179.6 (2)
C12—O13—C14—C15	−179.68 (16)	C131—C132—C133—C134	0.7 (5)
O13—C12—C121—C122	−93.5 (2)	C132—C133—C134—C135	−0.5 (5)
C131—C12—C121—C122	148.2 (2)	C133—C134—C135—C136	0.7 (4)
C11—C12—C121—C122	31.6 (3)	C132—C131—C136—C135	1.2 (4)
O13—C12—C121—C126	81.1 (2)	C12—C131—C136—C135	179.7 (2)
C131—C12—C121—C126	−37.2 (3)	C134—C135—C136—C131	−1.0 (4)

### N,N-Dimethyl-[2-(2,2-diphenyl)-2-prop-2-ynyloxyacetoxy]ethylammonium 2,4,6-trinitrophenolate (II) Crystal data

**Table:**

C_21_H_24_NO_3_·C_6_H_2_N_3_O_7_	*Z* = 2
*M**_r_* = 566.52	*F*(000) = 592
Triclinic, *P*1	*D*_x_ = 1.397 Mg m^−^^3^
*a* = 7.5208 (3) Å	Mo *K*α radiation, λ = 0.71073 Å
*b* = 8.3919 (3) Å	Cell parameters from 4348 reflections
*c* = 22.2282 (7) Å	θ = 2.4–27.6°
α = 85.099 (3)°	µ = 0.11 mm^−^^1^
β = 84.294 (3)°	*T* = 296 K
γ = 75.117 (3)°	Block, colourless
*V* = 1346.51 (9) Å^3^	0.23 × 0.21 × 0.21 mm

### N,N-Dimethyl-[2-(2,2-diphenyl)-2-prop-2-ynyloxyacetoxy]ethylammonium 2,4,6-trinitrophenolate (II) Data collection

**Table:**

Rigaku Saturn724 diffractometer	4973 reflections with *I* > 2σ(*I*)
Radiation source: fine focus sealed tube	*R*_int_ = 0.033
φ and ω scans	θ_max_ = 31.1°, θ_min_ = 2.5°
Absorption correction: multi-scan (SADABS; Sheldrick,2003)	*h* = −10→10
*T*_min_ = 0.949, *T*_max_ = 0.978	*k* = −12→9
18671 measured reflections	*l* = −31→31
7547 independent reflections	

### N,N-Dimethyl-[2-(2,2-diphenyl)-2-prop-2-ynyloxyacetoxy]ethylammonium 2,4,6-trinitrophenolate (II) Refinement

**Table:**

Refinement on *F*^2^	10 restraints
Least-squares matrix: full	Hydrogen site location: inferred from neighbouring sites
*R*[*F*^2^ > 2σ(*F*^2^)] = 0.053	H-atom parameters constrained
*wR*(*F*^2^) = 0.136	*w* = 1/[σ^2^(*F*_o_^2^) + (0.0499*P*)^2^ + 0.2103*P*] where *P* = (*F*_o_^2^ + 2*F*_c_^2^)/3
*S* = 1.05	(Δ/σ)_max_ = 0.001
7547 reflections	Δρ_max_ = 0.27 e Å^−^^3^
390 parameters	Δρ_min_ = −0.22 e Å^−^^3^

### N,N-Dimethyl-[2-(2,2-diphenyl)-2-prop-2-ynyloxyacetoxy]ethylammonium 2,4,6-trinitrophenolate (II) Special details

**Table:**

Geometry. All esds (except the esd in the dihedral angle between two l.s. planes) are estimated using the full covariance matrix. The cell esds are taken into account individually in the estimation of esds in distances, angles and torsion angles; correlations between esds in cell parameters are only used when they are defined by crystal symmetry. An approximate (isotropic) treatment of cell esds is used for estimating esds involving l.s. planes.

### N,N-Dimethyl-[2-(2,2-diphenyl)-2-prop-2-ynyloxyacetoxy]ethylammonium 2,4,6-trinitrophenolate (II) Fractional atomic coordinates and isotropic or equivalent isotropic displacement parameters (Å^2^)

**Table:**

	*x*	*y*	*z*	*U*_iso_*/*U*_eq_	Occ. (<1)
N1	0.6256 (10)	0.4825 (7)	0.6843 (3)	0.0517 (9)	0.654 (11)
H1	0.7502	0.4253	0.6689	0.062*	0.654 (11)
C111	0.5676 (10)	0.3670 (6)	0.7326 (4)	0.0770 (17)	0.654 (11)
H11A	0.5826	0.2612	0.7166	0.115*	0.654 (11)
H11B	0.4403	0.4105	0.7461	0.115*	0.654 (11)
H11C	0.6424	0.3550	0.7660	0.115*	0.654 (11)
C112	0.5108 (16)	0.5096 (11)	0.6316 (3)	0.0809 (18)	0.654 (11)
H11D	0.3834	0.5528	0.6451	0.121*	0.654 (11)
H11E	0.5252	0.4067	0.6136	0.121*	0.654 (11)
H11F	0.5499	0.5870	0.6022	0.121*	0.654 (11)
C1	0.6438 (8)	0.6401 (8)	0.7067 (4)	0.0500 (9)	0.654 (11)
H1A	0.7169	0.6903	0.6760	0.060*	0.654 (11)
H1B	0.7123	0.6133	0.7425	0.060*	0.654 (11)
C2	0.4667 (11)	0.7662 (7)	0.7221 (4)	0.0448 (9)	0.654 (11)
H2A	0.4931	0.8687	0.7308	0.054*	0.654 (11)
H2B	0.3907	0.7882	0.6881	0.054*	0.654 (11)
O11	0.36983 (14)	0.70288 (12)	0.77457 (5)	0.0435 (3)	0.654 (11)
N21	0.612 (2)	0.4863 (14)	0.6728 (5)	0.0517 (9)	0.346 (11)
H21	0.7335	0.4177	0.6599	0.062*	0.346 (11)
C211	0.5119 (18)	0.3703 (13)	0.7068 (6)	0.0770 (17)	0.346 (11)
H21A	0.3918	0.4310	0.7220	0.115*	0.346 (11)
H21B	0.5804	0.3152	0.7401	0.115*	0.346 (11)
H21C	0.4992	0.2901	0.6803	0.115*	0.346 (11)
C212	0.526 (3)	0.558 (2)	0.6156 (6)	0.0809 (18)	0.346 (11)
H21D	0.5839	0.6415	0.5975	0.121*	0.346 (11)
H21E	0.3966	0.6054	0.6245	0.121*	0.346 (11)
H21F	0.5424	0.4720	0.5881	0.121*	0.346 (11)
C21	0.6478 (14)	0.6122 (18)	0.7103 (7)	0.0500 (9)	0.346 (11)
H21G	0.7460	0.6556	0.6890	0.060*	0.346 (11)
H21H	0.6932	0.5566	0.7479	0.060*	0.346 (11)
C22	0.488 (2)	0.7555 (13)	0.7256 (7)	0.0448 (9)	0.346 (11)
H22A	0.5327	0.8455	0.7378	0.054*	0.346 (11)
H22B	0.4187	0.7945	0.6904	0.054*	0.346 (11)
O21	0.36983 (14)	0.70288 (12)	0.77457 (5)	0.0435 (3)	0.346 (11)
C11	0.2198 (2)	0.81409 (17)	0.79625 (6)	0.0359 (3)	
O12	0.16787 (16)	0.94998 (13)	0.77343 (5)	0.0506 (3)	
C12	0.13145 (19)	0.74885 (17)	0.85597 (6)	0.0335 (3)	
O13	−0.04211 (13)	0.86286 (12)	0.87024 (4)	0.0367 (2)	
C14	−0.1829 (2)	0.8619 (2)	0.83189 (8)	0.0481 (4)	
H14A	−0.2176	0.7577	0.8386	0.058*	
H14B	−0.1365	0.8725	0.7898	0.058*	
C15	−0.3429 (2)	0.9967 (2)	0.84437 (7)	0.0472 (4)	
C16	−0.4802 (3)	1.0991 (3)	0.85172 (10)	0.0723 (6)	
H16	−0.5894	1.1806	0.8576	0.087*	
C121	0.25642 (19)	0.75762 (17)	0.90515 (6)	0.0348 (3)	
C122	0.2015 (2)	0.87452 (19)	0.94821 (7)	0.0421 (4)	
H122	0.0868	0.9497	0.9473	0.051*	
C123	0.3161 (3)	0.8801 (2)	0.99240 (8)	0.0543 (4)	
H123	0.2772	0.9580	1.0214	0.065*	
C124	0.4869 (3)	0.7715 (3)	0.99388 (9)	0.0593 (5)	
H124	0.5638	0.7762	1.0236	0.071*	
C125	0.5439 (2)	0.6557 (2)	0.95124 (9)	0.0568 (5)	
H125	0.6600	0.5826	0.9519	0.068*	
C126	0.4292 (2)	0.6476 (2)	0.90731 (8)	0.0471 (4)	
H126	0.4680	0.5678	0.8790	0.056*	
C131	0.1025 (2)	0.57518 (17)	0.85444 (6)	0.0357 (3)	
C132	0.0746 (2)	0.48827 (19)	0.90896 (7)	0.0443 (4)	
H132	0.0871	0.5313	0.9451	0.053*	
C133	0.0288 (3)	0.3393 (2)	0.91052 (8)	0.0537 (4)	
H133	0.0097	0.2832	0.9475	0.064*	
C134	0.0110 (3)	0.2730 (2)	0.85755 (9)	0.0540 (4)	
H134	−0.0201	0.1724	0.8587	0.065*	
C135	0.0395 (2)	0.3566 (2)	0.80287 (8)	0.0497 (4)	
H135	0.0295	0.3116	0.7669	0.060*	
C136	0.0830 (2)	0.50791 (19)	0.80123 (7)	0.0424 (4)	
H136	0.0994	0.5648	0.7642	0.051*	
C31	1.0191 (2)	0.18823 (19)	0.61774 (7)	0.0424 (4)	
O31	0.93492 (18)	0.25416 (15)	0.66415 (5)	0.0584 (3)	
C32	1.0549 (2)	0.01354 (19)	0.60957 (7)	0.0426 (4)	
N32	0.9763 (2)	−0.08842 (19)	0.65583 (6)	0.0529 (4)	
O32	0.8237 (2)	−0.03049 (19)	0.68053 (7)	0.0753 (4)	
O33	1.0676 (2)	−0.22946 (16)	0.66751 (6)	0.0688 (4)	
C33	1.1556 (2)	−0.0640 (2)	0.56099 (7)	0.0450 (4)	
H33	1.1750	−0.1773	0.5585	0.054*	
C34	1.2283 (2)	0.0300 (2)	0.51549 (7)	0.0435 (4)	
N34	1.3347 (2)	−0.0478 (2)	0.46332 (7)	0.0558 (4)	
O34	1.3586 (2)	−0.19701 (19)	0.46114 (7)	0.0872 (5)	
O35	1.3986 (2)	0.0377 (2)	0.42408 (6)	0.0740 (4)	
C35	1.2027 (2)	0.1978 (2)	0.51881 (7)	0.0438 (4)	
H35	1.2539	0.2590	0.4882	0.053*	
C36	1.1010 (2)	0.27324 (19)	0.56788 (7)	0.0429 (4)	
N36	1.0748 (2)	0.45097 (18)	0.56790 (7)	0.0525 (4)	
O36	1.1884 (2)	0.51340 (17)	0.53894 (7)	0.0749 (4)	
O37	0.9371 (2)	0.53363 (16)	0.59497 (7)	0.0786 (5)	

### N,N-Dimethyl-[2-(2,2-diphenyl)-2-prop-2-ynyloxyacetoxy]ethylammonium 2,4,6-trinitrophenolate (II) Atomic displacement parameters (Å^2^)

**Table:**

	*U*^11^	*U*^22^	*U*^33^	*U*^12^	*U*^13^	*U*^23^
N1	0.0486 (13)	0.0446 (9)	0.057 (2)	−0.0052 (9)	0.0087 (16)	−0.0119 (13)
C111	0.074 (4)	0.0484 (13)	0.099 (4)	−0.012 (2)	0.027 (3)	−0.006 (3)
C112	0.080 (2)	0.080 (5)	0.082 (4)	−0.004 (3)	−0.015 (4)	−0.038 (3)
C1	0.0448 (9)	0.050 (2)	0.0532 (12)	−0.0107 (12)	0.0077 (9)	−0.0113 (16)
C2	0.048 (2)	0.0416 (11)	0.0435 (12)	−0.0126 (11)	0.0092 (14)	−0.0078 (8)
O11	0.0461 (6)	0.0387 (6)	0.0399 (5)	−0.0045 (5)	0.0090 (5)	−0.0042 (4)
N21	0.0486 (13)	0.0446 (9)	0.057 (2)	−0.0052 (9)	0.0087 (16)	−0.0119 (13)
C211	0.074 (4)	0.0484 (13)	0.099 (4)	−0.012 (2)	0.027 (3)	−0.006 (3)
C212	0.080 (2)	0.080 (5)	0.082 (4)	−0.004 (3)	−0.015 (4)	−0.038 (3)
C21	0.0448 (9)	0.050 (2)	0.0532 (12)	−0.0107 (12)	0.0077 (9)	−0.0113 (16)
C22	0.048 (2)	0.0416 (11)	0.0435 (12)	−0.0126 (11)	0.0092 (14)	−0.0078 (8)
O21	0.0461 (6)	0.0387 (6)	0.0399 (5)	−0.0045 (5)	0.0090 (5)	−0.0042 (4)
C11	0.0409 (7)	0.0302 (7)	0.0364 (7)	−0.0080 (6)	0.0004 (6)	−0.0075 (6)
O12	0.0616 (7)	0.0322 (6)	0.0510 (6)	−0.0049 (5)	0.0082 (6)	0.0000 (5)
C12	0.0359 (7)	0.0290 (7)	0.0339 (7)	−0.0051 (6)	0.0007 (6)	−0.0065 (5)
O13	0.0348 (5)	0.0349 (5)	0.0388 (5)	−0.0034 (4)	−0.0026 (4)	−0.0105 (4)
C14	0.0429 (8)	0.0521 (10)	0.0487 (9)	−0.0054 (7)	−0.0086 (7)	−0.0133 (7)
C15	0.0421 (8)	0.0545 (10)	0.0431 (8)	−0.0085 (8)	−0.0046 (7)	−0.0029 (7)
C16	0.0510 (11)	0.0828 (15)	0.0684 (13)	0.0096 (10)	−0.0043 (10)	−0.0039 (11)
C121	0.0382 (7)	0.0324 (7)	0.0353 (7)	−0.0126 (6)	0.0000 (6)	−0.0021 (5)
C122	0.0470 (8)	0.0407 (8)	0.0404 (8)	−0.0128 (7)	−0.0029 (7)	−0.0075 (6)
C123	0.0671 (11)	0.0556 (11)	0.0463 (9)	−0.0224 (9)	−0.0085 (8)	−0.0102 (8)
C124	0.0611 (11)	0.0720 (13)	0.0535 (10)	−0.0282 (10)	−0.0186 (9)	0.0002 (9)
C125	0.0436 (9)	0.0635 (12)	0.0617 (11)	−0.0107 (8)	−0.0124 (8)	0.0069 (9)
C126	0.0444 (8)	0.0451 (9)	0.0489 (9)	−0.0054 (7)	−0.0041 (7)	−0.0047 (7)
C131	0.0365 (7)	0.0306 (7)	0.0389 (7)	−0.0065 (6)	−0.0001 (6)	−0.0060 (6)
C132	0.0551 (9)	0.0370 (8)	0.0417 (8)	−0.0146 (7)	0.0026 (7)	−0.0051 (6)
C133	0.0647 (11)	0.0384 (9)	0.0575 (10)	−0.0171 (8)	0.0051 (9)	0.0012 (7)
C134	0.0572 (10)	0.0334 (8)	0.0741 (12)	−0.0151 (8)	−0.0045 (9)	−0.0078 (8)
C135	0.0547 (10)	0.0379 (9)	0.0589 (10)	−0.0094 (8)	−0.0123 (8)	−0.0148 (7)
C136	0.0496 (9)	0.0359 (8)	0.0419 (8)	−0.0091 (7)	−0.0057 (7)	−0.0058 (6)
C31	0.0410 (8)	0.0420 (8)	0.0398 (8)	−0.0002 (7)	−0.0074 (6)	−0.0044 (6)
O31	0.0670 (8)	0.0537 (7)	0.0424 (6)	0.0041 (6)	0.0043 (6)	−0.0061 (5)
C32	0.0416 (8)	0.0405 (8)	0.0443 (8)	−0.0074 (7)	−0.0084 (7)	0.0011 (6)
N32	0.0600 (9)	0.0550 (9)	0.0464 (8)	−0.0180 (8)	−0.0134 (7)	0.0032 (6)
O32	0.0622 (9)	0.0881 (11)	0.0710 (9)	−0.0208 (8)	0.0053 (7)	0.0144 (8)
O33	0.0942 (10)	0.0463 (7)	0.0640 (8)	−0.0138 (7)	−0.0185 (7)	0.0094 (6)
C33	0.0454 (8)	0.0371 (8)	0.0520 (9)	−0.0041 (7)	−0.0133 (7)	−0.0073 (7)
C34	0.0393 (8)	0.0450 (9)	0.0441 (8)	−0.0031 (7)	−0.0043 (7)	−0.0124 (7)
N34	0.0474 (8)	0.0617 (10)	0.0562 (9)	−0.0045 (7)	−0.0035 (7)	−0.0219 (8)
O34	0.0967 (12)	0.0629 (9)	0.0981 (12)	−0.0099 (8)	0.0170 (9)	−0.0424 (8)
O35	0.0716 (9)	0.0897 (11)	0.0557 (8)	−0.0140 (8)	0.0126 (7)	−0.0159 (7)
C35	0.0423 (8)	0.0445 (9)	0.0428 (8)	−0.0071 (7)	−0.0033 (7)	−0.0037 (6)
C36	0.0465 (8)	0.0358 (8)	0.0451 (8)	−0.0061 (7)	−0.0056 (7)	−0.0057 (6)
N36	0.0639 (9)	0.0414 (8)	0.0504 (8)	−0.0098 (7)	−0.0026 (7)	−0.0064 (6)
O36	0.0896 (10)	0.0533 (8)	0.0851 (10)	−0.0293 (8)	0.0079 (9)	−0.0052 (7)
O37	0.0959 (11)	0.0432 (7)	0.0824 (10)	0.0016 (7)	0.0185 (8)	−0.0132 (7)

### N,N-Dimethyl-[2-(2,2-diphenyl)-2-prop-2-ynyloxyacetoxy]ethylammonium 2,4,6-trinitrophenolate (II) Geometric parameters (Å, º)

**Table:**

N1—C111	1.492 (3)	C16—H16	0.9300
N1—C112	1.492 (4)	C121—C122	1.387 (2)
N1—C1	1.495 (3)	C121—C126	1.390 (2)
N1—H1	0.9800	C122—C123	1.381 (2)
C111—H11A	0.9600	C122—H122	0.9300
C111—H11B	0.9600	C123—C124	1.373 (3)
C111—H11C	0.9600	C123—H123	0.9300
C112—H11D	0.9600	C124—C125	1.374 (3)
C112—H11E	0.9600	C124—H124	0.9300
C112—H11F	0.9600	C125—C126	1.383 (2)
C1—C2	1.503 (3)	C125—H125	0.9300
C1—H1A	0.9700	C126—H126	0.9300
C1—H1B	0.9700	C131—C132	1.386 (2)
C2—O11	1.447 (3)	C131—C136	1.389 (2)
C2—H2A	0.9700	C132—C133	1.376 (2)
C2—H2B	0.9700	C132—H132	0.9300
O11—C11	1.3432 (16)	C133—C134	1.377 (3)
N21—C211	1.489 (5)	C133—H133	0.9300
N21—C212	1.490 (5)	C134—C135	1.377 (3)
N21—C21	1.493 (4)	C134—H134	0.9300
N21—H21	0.9800	C135—C136	1.387 (2)
C211—H21A	0.9600	C135—H135	0.9300
C211—H21B	0.9600	C136—H136	0.9300
C211—H21C	0.9600	C31—O31	1.2486 (17)
C212—H21D	0.9600	C31—C36	1.439 (2)
C212—H21E	0.9600	C31—C32	1.445 (2)
C212—H21F	0.9600	C32—C33	1.367 (2)
C21—C22	1.502 (4)	C32—N32	1.459 (2)
C21—H21G	0.9700	N32—O32	1.220 (2)
C21—H21H	0.9700	N32—O33	1.2267 (19)
C22—H22A	0.9700	C33—C34	1.385 (2)
C22—H22B	0.9700	C33—H33	0.9300
C11—O12	1.1925 (17)	C34—C35	1.379 (2)
C11—C12	1.546 (2)	C34—N34	1.445 (2)
C12—O13	1.4311 (15)	N34—O35	1.222 (2)
C12—C121	1.529 (2)	N34—O34	1.223 (2)
C12—C131	1.532 (2)	C35—C36	1.371 (2)
O13—C14	1.4261 (19)	C35—H35	0.9300
C14—C15	1.447 (2)	C36—N36	1.453 (2)
C14—H14A	0.9700	N36—O36	1.2170 (19)
C14—H14B	0.9700	N36—O37	1.2226 (18)
C15—C16	1.169 (2)		
			
C111—N1—C112	112.0 (3)	C15—C14—H14A	109.6
C111—N1—C1	114.3 (3)	O13—C14—H14B	109.6
C112—N1—C1	112.9 (3)	C15—C14—H14B	109.6
C111—N1—H1	105.6	H14A—C14—H14B	108.1
C112—N1—H1	105.6	C16—C15—C14	174.3 (2)
C1—N1—H1	105.6	C15—C16—H16	180.0
N1—C111—H11A	109.5	C122—C121—C126	118.53 (14)
N1—C111—H11B	109.5	C122—C121—C12	121.32 (13)
H11A—C111—H11B	109.5	C126—C121—C12	120.15 (13)
N1—C111—H11C	109.5	C123—C122—C121	120.45 (15)
H11A—C111—H11C	109.5	C123—C122—H122	119.8
H11B—C111—H11C	109.5	C121—C122—H122	119.8
N1—C112—H11D	109.5	C124—C123—C122	120.53 (16)
N1—C112—H11E	109.5	C124—C123—H123	119.7
H11D—C112—H11E	109.5	C122—C123—H123	119.7
N1—C112—H11F	109.5	C123—C124—C125	119.72 (17)
H11D—C112—H11F	109.5	C123—C124—H124	120.1
H11E—C112—H11F	109.5	C125—C124—H124	120.1
N1—C1—C2	116.3 (3)	C124—C125—C126	120.22 (17)
N1—C1—H1A	108.2	C124—C125—H125	119.9
C2—C1—H1A	108.2	C126—C125—H125	119.9
N1—C1—H1B	108.2	C125—C126—C121	120.53 (15)
C2—C1—H1B	108.2	C125—C126—H126	119.7
H1A—C1—H1B	107.4	C121—C126—H126	119.7
O11—C2—C1	108.8 (3)	C132—C131—C136	118.23 (14)
O11—C2—H2A	109.9	C132—C131—C12	118.48 (13)
C1—C2—H2A	109.9	C136—C131—C12	122.91 (13)
O11—C2—H2B	109.9	C133—C132—C131	121.04 (15)
C1—C2—H2B	109.9	C133—C132—H132	119.5
H2A—C2—H2B	108.3	C131—C132—H132	119.5
C11—O11—C2	113.4 (3)	C132—C133—C134	120.36 (16)
C211—N21—C212	111.9 (6)	C132—C133—H133	119.8
C211—N21—C21	114.3 (6)	C134—C133—H133	119.8
C212—N21—C21	113.7 (5)	C133—C134—C135	119.54 (16)
C211—N21—H21	105.3	C133—C134—H134	120.2
C212—N21—H21	105.3	C135—C134—H134	120.2
C21—N21—H21	105.3	C134—C135—C136	120.19 (16)
N21—C211—H21A	109.5	C134—C135—H135	119.9
N21—C211—H21B	109.5	C136—C135—H135	119.9
H21A—C211—H21B	109.5	C135—C136—C131	120.62 (15)
N21—C211—H21C	109.5	C135—C136—H136	119.7
H21A—C211—H21C	109.5	C131—C136—H136	119.7
H21B—C211—H21C	109.5	O31—C31—C36	124.73 (15)
N21—C212—H21D	109.5	O31—C31—C32	123.31 (15)
N21—C212—H21E	109.5	C36—C31—C32	111.87 (13)
H21D—C212—H21E	109.5	C33—C32—C31	124.78 (15)
N21—C212—H21F	109.5	C33—C32—N32	117.05 (14)
H21D—C212—H21F	109.5	C31—C32—N32	118.17 (14)
H21E—C212—H21F	109.5	O32—N32—O33	123.34 (16)
N21—C21—C22	116.8 (6)	O32—N32—C32	118.84 (15)
N21—C21—H21G	108.1	O33—N32—C32	117.82 (15)
C22—C21—H21G	108.1	C32—C33—C34	118.50 (15)
N21—C21—H21H	108.1	C32—C33—H33	120.8
C22—C21—H21H	108.1	C34—C33—H33	120.8
H21G—C21—H21H	107.3	C35—C34—C33	121.46 (14)
C21—C22—H22A	109.9	C35—C34—N34	118.57 (15)
C21—C22—H22B	109.9	C33—C34—N34	119.97 (15)
H22A—C22—H22B	108.3	O35—N34—O34	123.21 (15)
O12—C11—O11	123.37 (13)	O35—N34—C34	118.67 (15)
O12—C11—C12	124.45 (13)	O34—N34—C34	118.12 (17)
O11—C11—C12	112.07 (11)	C36—C35—C34	119.12 (15)
O13—C12—C121	106.58 (10)	C36—C35—H35	120.4
O13—C12—C131	109.47 (11)	C34—C35—H35	120.4
C121—C12—C131	111.76 (11)	C35—C36—C31	124.26 (14)
O13—C12—C11	108.04 (11)	C35—C36—N36	116.46 (15)
C121—C12—C11	105.98 (11)	C31—C36—N36	119.27 (13)
C131—C12—C11	114.62 (11)	O36—N36—O37	122.06 (15)
C14—O13—C12	114.74 (10)	O36—N36—C36	118.51 (14)
O13—C14—C15	110.22 (13)	O37—N36—C36	119.38 (15)
O13—C14—H14A	109.6		
			
C111—N1—C1—C2	−75.6 (5)	C11—C12—C131—C136	−25.2 (2)
C112—N1—C1—C2	54.0 (6)	C136—C131—C132—C133	0.0 (2)
N1—C1—C2—O11	67.3 (7)	C12—C131—C132—C133	173.15 (15)
C1—C2—O11—C11	172.8 (4)	C131—C132—C133—C134	0.5 (3)
C211—N21—C21—C22	−77.1 (10)	C132—C133—C134—C135	0.1 (3)
C212—N21—C21—C22	53.1 (12)	C133—C134—C135—C136	−1.0 (3)
C2—O11—C11—O12	2.3 (5)	C134—C135—C136—C131	1.4 (3)
C2—O11—C11—C12	−174.0 (5)	C132—C131—C136—C135	−0.9 (2)
O12—C11—C12—O13	13.1 (2)	C12—C131—C136—C135	−173.77 (14)
O11—C11—C12—O13	−170.68 (11)	O31—C31—C32—C33	−176.05 (16)
O12—C11—C12—C121	−100.86 (17)	C36—C31—C32—C33	0.6 (2)
O11—C11—C12—C121	75.41 (14)	O31—C31—C32—N32	4.9 (2)
O12—C11—C12—C131	135.39 (16)	C36—C31—C32—N32	−178.42 (13)
O11—C11—C12—C131	−48.34 (17)	C33—C32—N32—O32	−142.86 (17)
C121—C12—O13—C14	−174.95 (12)	C31—C32—N32—O32	36.3 (2)
C131—C12—O13—C14	−53.92 (15)	C33—C32—N32—O33	36.5 (2)
C11—C12—O13—C14	71.53 (15)	C31—C32—N32—O33	−144.32 (16)
C12—O13—C14—C15	−171.72 (13)	C31—C32—C33—C34	−0.7 (2)
O13—C12—C121—C122	−7.05 (18)	N32—C32—C33—C34	178.39 (14)
C131—C12—C121—C122	−126.61 (14)	C32—C33—C34—C35	0.8 (2)
C11—C12—C121—C122	107.87 (15)	C32—C33—C34—N34	−179.70 (14)
O13—C12—C121—C126	173.18 (13)	C35—C34—N34—O35	0.1 (2)
C131—C12—C121—C126	53.63 (17)	C33—C34—N34—O35	−179.37 (15)
C11—C12—C121—C126	−71.90 (16)	C35—C34—N34—O34	179.07 (16)
C126—C121—C122—C123	−0.5 (2)	C33—C34—N34—O34	−0.4 (2)
C12—C121—C122—C123	179.77 (14)	C33—C34—C35—C36	−0.9 (2)
C121—C122—C123—C124	0.9 (3)	N34—C34—C35—C36	179.57 (15)
C122—C123—C124—C125	−0.4 (3)	C34—C35—C36—C31	0.9 (2)
C123—C124—C125—C126	−0.6 (3)	C34—C35—C36—N36	−178.34 (14)
C124—C125—C126—C121	1.0 (3)	O31—C31—C36—C35	175.86 (16)
C122—C121—C126—C125	−0.4 (2)	C32—C31—C36—C35	−0.8 (2)
C12—C121—C126—C125	179.32 (15)	O31—C31—C36—N36	−4.9 (2)
O13—C12—C131—C132	−76.50 (16)	C32—C31—C36—N36	178.50 (14)
C121—C12—C131—C132	41.34 (17)	C35—C36—N36—O36	−25.2 (2)
C11—C12—C131—C132	161.95 (13)	C31—C36—N36—O36	155.44 (16)
O13—C12—C131—C136	96.35 (16)	C35—C36—N36—O37	152.17 (16)
C121—C12—C131—C136	−145.81 (14)	C31—C36—N36—O37	−27.2 (2)

### N,N-Dimethyl-[2-(2,2-diphenyl)-2-prop-2-ynyloxyacetoxy]ethylammonium 2,4,6-trinitrophenolate (II) Hydrogen-bond geometry (Å, º)

**Table:**

*D*—H···*A*	*D*—H	H···*A*	*D*···*A*	*D*—H···*A*
N1—H1···O31	0.98	1.72	2.633 (7)	153
N1—H1···O37	0.98	2.33	3.010 (7)	126
N21—H21···O31	0.98	1.77	2.697 (14)	157
N21—H21···O37	0.98	2.34	2.944 (14)	120
C2—H2*B*···O33^i^	0.97	2.56	3.338 (9)	137
C14—H14*B*···O32^i^	0.97	2.52	3.407 (2)	153
C135—H135···O31^ii^	0.93	2.58	3.485 (2)	165

Symmetry codes: (i) *x*−1, *y*+1, *z*; (ii) *x*−1, *y*, *z*.
